# Serum phosphate concentration during the rewarming period after deep hypothermic circulatory arrest

**DOI:** 10.1186/cc13691

**Published:** 2014-03-17

**Authors:** H Hayami, O Yamaguchi, S Ishida, Y Koide, T Gotoh

**Affiliations:** 1 Yokohama Municipal Citizen’s Hospital, Yokohama, Japan; 2 Yokohama City University Hospital, Yokohama, Japan; 3 Yokohama City University Medical Center, Yokohama, Japan; 4 Hayama Heart Center, Hayama, Japan

## Introduction

Short-term neuropsychologic dysfunction (SND) is often observed during the postoperative period of open total arch repair (TAR) under deep hypothermic circulatory arrest (DHCA). We supposed that there might be a large consumption of high-energy phosphates during the rewarming period, and this might be associated with awakening and SND. Due to difficulty in measuring such high-energy phosphates at the bedside, we measured the serum phosphate concentration (P) as a substitute, and assessed its influence on awakening and postoperative SND.

## Methods

Twenty patients with a mean age of 68 ± 11 years who underwent open TAR under DHCA applied at a temperature of 20°C, with antegrade cerebral perfusion (ACP) or retrograde cerebral perfusion (RCP), were enrolled. Arterial blood gas lactate concentration (ALac), ScvO_2_, and P were measured at 2, 4, 6, and 10 hours after weaning from cardiopulmonary bypass (CPB). We also measured time to confirm M6 on the Glasgow Coma Scale (GCS), and incidence of SND.

Occurrence of SND was defined as neurological disarrangement such as irritability, confusion, or delirium within 48 hours after operation.

## Results

Durations of CPB, aortic clamp, and circulatory arrest were 194 ± 51, 136 ± 53, and 70 ± 17 minutes respectively. Nine patients underwent ACP (89 ± 26 minutes) and 11 patients underwent RCP (56 ± 25 minutes). Concentration of ALac, ScvO_2_, and P changed at 2, 4, 6, 10 hours after CPB as follows: ALac 4.0 ± 1.9, 5.1 ± 2.6, 5.3 ± 3.3, 4.0 ± 2.8 mmol/l, ScvO_2 _71 ± 6.7, 67 ± 10, 67 ± 9.7, 65 ± 11%, and P 2.9 ± 0.9, 2.4 ± 0.7, 2.2 ± 1.0, 1.8 ± 0.8 mg/dl, respectively. Serum P recovered to 2.6 ± 1.4 at 18 hours after CPB. The incidence of hypophosphatemia (<2.6 mg/dl) and SND in our series were 18/20 (90%) and 14/20(70%) respectively. There was a correlation between minimum P and time to confirm M6 on the GCS (*P *= 0.508, Fisher r-to-z transformation), but no significant correlation between SND. The mortality rate during the first 28 days was 5% (1/20).

## Conclusion

Serum phosphate decreased dramatically during the rewarming period after deep hypothermic circulatory arrest. There was a moderate correlation between minimum phosphate concentration and time to confirm M6 on the GCS. Monitoring phosphate concentration might predict neurological recovery after deep hypothermic circulatory arrest.

